# Giant solitary fibrous tumor of the pelvis successfully treated with preoperative embolization and surgical resection: a case report

**DOI:** 10.1186/s12957-015-0578-6

**Published:** 2015-04-29

**Authors:** Yuichiro Yokoyama, Keisuke Hata, Takamitsu Kanazawa, Hironori Yamaguchi, Soichiro Ishihara, Eiji Sunami, Joji Kitayama, Toshiaki Watanabe

**Affiliations:** Division of Surgical Oncology, Department of Surgery, Faculty of Medicine, The University of Tokyo, 7-3-1 Hongo, Bunkyo-ku, Tokyo 113-8655 Japan

**Keywords:** Solitary fibrous tumor, Embolization, Pelvis

## Abstract

Solitary fibrous tumors (SFTs) rarely develop in the pelvis. When they do arise, they are usually treated using surgery, although SFTs are often very large by the time of diagnosis, which makes surgical excision difficult. We report a case of a 63-year-old man who was referred to our hospital for the treatment of a giant tumor of the pelvis. Computed tomography (CT) revealed a 30 × 25 × 19 cm sized hypervascular tumor that almost completely filled the pelvic cavity. The diagnosis of SFT was made by CT-assisted needle biopsy. The feeding arteries of the tumor were embolized twice. The first embolization aimed to reduce the tumor volume, while the second one was planned a day prior to the surgery to obtain hematostasis during the operation. Tumor resection was then performed. The blood loss during the operation was 440 ml, and there was no uncontrollable bleeding. The postoperative course was uneventful. No recurrence of SFT was observed during a 2-year follow-up.

## Background

Solitary fibrous tumor (SFT) is a rare mesenchymal tumor that was first documented by Klemperer and Rabin [1]. Although SFTs mainly develop in the pleura, they have also been found in various extra-thoracic sites, such as the peritoneum, liver, thymus, thyroid, intrapulmonary parenchyma, and nasal cavity [2]. Thirty percent of SFTs occur in the abdominopelvic region, making it one of the major primary sites for this tumor [3]. Surgical resection is the treatment of choice for SFTs. However, abdominopelvic SFTs are often very large by the time of diagnosis, which makes surgical resection difficult. Because of their rarity, the preoperative management for the safe resection of abdominopelvic SFTs has not been fully defined. Here, we report a case of a giant pelvic SFT that was successfully resected following preoperative embolization of the feeding arteries.

## Case presentation

**Figure 1:**
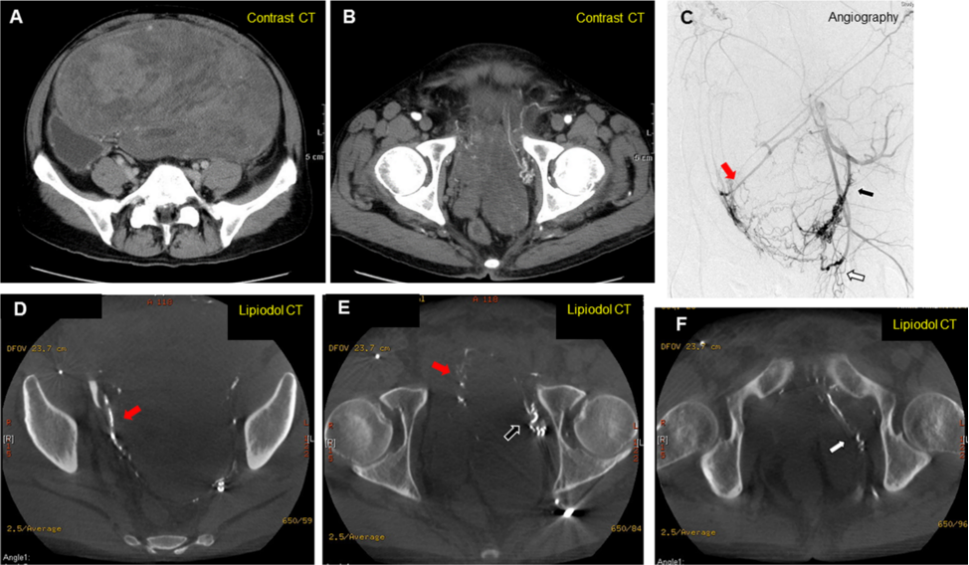
Preoperative computed tomography (CT), angiography before embolization, and plain CT after embolization with lipiodol and *N*-butyl-2-cyanoacrylate. **(A)**, **(B)** Preoperative CT showed a giant pelvic mass lesion, measuring 30 × 25 × 19 cm. **(C)** Angiography of the left iliac artery revealed that the mass was mainly supplied by the left obturator artery (black arrow), left internal pudendal artery (white arrow), and left superior vesical artery (red arrow). **(D)**, **(E)**, **(F)** CT after embolization showed that lipiodol and *N*-butyl-2-cyanoacrylate were well distributed to the left obturator artery (black arrow), left internal pudendal artery (white arrow), and left superior vesical artery (red arrow), demonstrating successful embolization of the target vessels.

**Figure 2:**
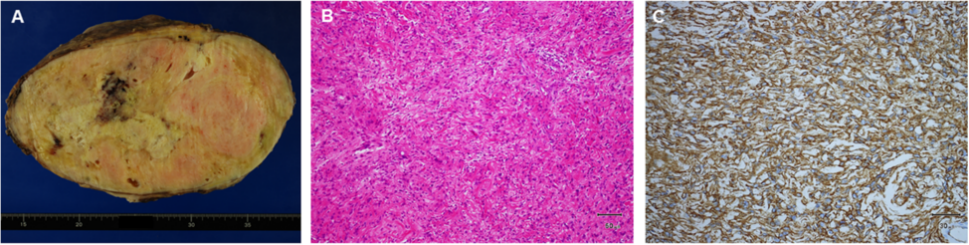
Macroscopic, microscopic, and immunohistological tumor findings. **(A)** Gross appearance of the resected tumor. **(B)** Microscopic findings were of a disordered arrangement of spindle cells (×100, original magnification). **(C)** Immunohistochemical staining revealed that tumor cells were positive for CD34 (×200, original magnification).

A 63-year-old man presented to another hospital with frequent urination was diagnosed as having a very large pelvic tumor. He was referred to our hospital for further treatment. He had a history of medical treatment for diabetes from the age of 53. Physical examination showed a large painless mass in the lower abdomen. Laboratory examination, including tumor markers, revealed no abnormalities except for a positive treadmill exercise test. Computed tomography (CT) and magnetic resonance imaging (MRI) revealed a huge mass, measuring 30 × 25 × 19 cm, which occupied the pelvic cavity and was suggestive of a malignant mesenchymal tumor (Figure 1A,B). The size of the tumor had increased by 20% in 2 months since he was first diagnosed at the other hospital. Angiography revealed a hypervascular tumor fed mainly by the left obturator artery, left internal pudendal artery, and left superior vesical artery (Figure 1C). CT-assisted needle biopsy was conducted to enable a pathological diagnosis. Histopathological examination revealed CD34-positive proliferating spindle cells in a collagenous matrix, suggestive of SFT. Coronary angiography showed total occlusion of the left anterior descending coronary artery, hypoplasia of the left circumflex coronary artery, and 50% stenosis of the right coronary artery. Percutaneous coronary intervention was therefore scheduled postoperatively. In order to reduce the tumor volume, the feeding arteries of the tumor were embolized. However, 1 month after the first embolization, CT revealed that the tumor had not reduced in size. Elective surgery was planned, and the second embolization of feeding arteries with lipiodol and *N*-butyl-2-cyanoacrylate was conducted a day prior to the operation to prevent uncontrollable bleeding (Figure 1D,E,F). Laparotomy was then performed through a midline incision. The tumor totally occupied the pelvic cavity. It seemed to have grown expansively and was grossly well circumscribed, but it had also partly adhered to the left pelvic wall. Its surface was covered by a plexiform meshwork of large dilated vessels that were susceptible to hemorrhage. Vessels entering the tumor were carefully ligated. The tumor did not invade the rectum or bladder and could be safely resected without massive bleeding. The needle biopsy tract was not resected. The total blood loss was 440 ml and no blood transfusion was needed. The resected tumor measured 30 × 25 × 15 cm. Macroscopically, the resected specimen was an encapsulated mass with focal hemorrhages and necrosis. The cut surface was solid and grayish-white (Figure 2A). Microscopic findings were of proliferating round and spindle cells, which exhibited a disordered arrangement in a collagenous matrix without significant cytological atypia (Figure 2B). The mitosis rate was low (<1/10 high-power fields), and only 1% of tumor cells were MIB-1 positive. Immunohistochemical staining demonstrated that the tumor cells were positive for CD34, vimentin, and Bcl-2, and negative for Desmin, S-100, and c-kit (Figure 2C), which was diagnostic for SFT. The postoperative course was uneventful, and no recurrence was apparent two years after surgery.

## Discussion

SFT was first described by Klemperer and Rabin in 1931 as a localized fibrous mesothelioma, but it is now recognized that SFTs can originate in various extra-thoracic sites. The age of onset of SFTs is around 50 to 60 years, and the male-to-female ratio appears to be almost equal [4]. Surgical resection is the treatment of choice for both abdominopelvic SFTs and those that arise in other organs. No standard therapy has been established for inoperable SFTs, and their surgical resectability is the most important prognostic factor [5], as complete resection of the tumor is curative in more than 90% of cases. However, abdominopelvic SFTs often remain asymptomatic and undiagnosed until they are very large. Indeed, Wang et al. reported that the diameters of abdominopelvic SFTs ranged between 2.5 and 28 cm (mean, 12.7 cm), which sometimes makes surgical resection difficult. In our case, the maximum diameter of the tumor was larger than in previous reports [2-4]. In general, neoadjuvant therapies were applied for unresectable gigantic tumors after a preoperative diagnosis was made. However, because of their rarity, the preoperative management of abdominopelvic SFTs has not been defined.

In the present case, CT revealed a giant mass that filled the pelvic cavity. The preoperative assessment revealed the patient had ischemic heart disease. To resect this tumor safely, the preoperative diagnosis was vital in order to determine the appropriate neoadjuvant therapy. On CT and MRI scans, SFTs are usually heterogeneous, with hypervascular areas showing intense enhancement, hypercellular areas showing moderate enhancement, and areas of cystic, myxoid degeneration, or necrosis showing no enhancement. However, these imaging characteristics are also observed in other hypervascular tumors with fibrous content, including most sarcomas, gastrointestinal stroma tumors, and malignant peripheral nerve sheath tumors, and hence, it is difficult to differentiate these tumors by CT and MRI findings alone [4]. Fluorodeoxyglucose positron-emission tomography shows heterogeneous accumulation of fluorodeoxyglucose, and ultrasonography reveals a heterogeneous echotexture tumor; however, these are not specific features for SFTs [6]. Indeed, in our case, enhancing CT could only indicate that this tumor was possibly a malignant mesenchymal tumor, and it did not allow a definitive diagnosis. These indistinct imaging findings explain why abdominopelvic SFTs are diagnosed postoperatively in most cases [3]. As we were not able to make a diagnosis based on CT findings, we performed a needle biopsy. The risk associated with a percutaneous core biopsy seemed to be minimal, and only a few cases of tumor seeding of sarcomas after this procedure have been reported [7-9]. Therefore, a needle biopsy was taken in order to obtain a preoperative diagnosis, which allowed a definitive diagnosis of SFT to be made.

The reported success of neoadjuvant therapy for SFTs has varied. Combs et al. reported that radiotherapy was effective in controlling SFTs of the central nervous system and spine, and some good results with radiotherapy have also been reported for abdominopelvic SFTs [10-13]. On this basis, irradiation is a candidate neoadjuvant therapy, although other studies have given disappointing results, and the efficacy of radiotherapy for SFTs continues to be debated [3]. Adriamycin or a combination of cyclophosphamide, vincristine, doxorubicin, and dacarbazine have been considered to be the most effective chemotherapeutic treatments for SFTs, but they have not been shown to prolong overall survival [12,13].

Unlike the approached described above, preoperative percutaneous embolization of feeding arteries has been shown to allow a safe, complete resection to be performed, especially in cases of thoracic, cervical, and spinal SFTs [14-16]. In the case of abdominopelvic SFTs, a few reports have described but not emphasized the use of preoperative embolization, and most other reports have not discussed its importance [6,17]. The resection of giant pelvic tumors, even if they are benign, is usually complicated by difficulties in visualizing the operative field, and the resection of giant pelvic hypervascular tumors such as SFTs is sometimes very dangerous. Indeed, a massive transfusion was needed in several cases of abdominopelvic SFT resection, and in two cases, patients died postoperatively of hemorrhagic shock [18,19]. In our case, angiography revealed a hypervascular tumor that was fed by branches of the left internal iliac artery. Hence, embolization of the feeding arteries was performed to shrink the tumor and to prevent bleeding during the operation. Potential severe complications of embolization are related to ischemia and arterial occlusion. These complications have been reported in the stomach, spleen, and kidney, but they rarely occur in the pelvis [20]. Unfortunately, the first embolization failed to reduce the tumor volume, although it prevented further tumor growth. The second embolization was successfully performed, leading to a safe resection without blood transfusion. In our case, the size of tumor was larger than that of previous reports, nevertheless the postoperative course was uneventful, and no recurrence was observed 2 years after surgery.

## Conclusion

Preoperative diagnosis and the embolization of feeding arteries are essential for the successful complete surgical resection of giant abdominopelvic SFTs.

## Consent

Written informed consent was obtained from the patient for publication of this case report and any accompanying images. A copy of this written consent is available for review by the Editor-in-Chief of this journal.

## Abbreviations

SFTs: solitary fibrous tumors
CT: computed tomography
